# How Many Diet-Related Non-Communicable Disease Deaths Could Be Averted or Delayed If Canadians Reduced Their Consumption of Calories Derived from Free Sugars Intake? A Macrosimulation Modeling Study

**DOI:** 10.3390/nu15081835

**Published:** 2023-04-11

**Authors:** Nadia Flexner, Jodi T. Bernstein, Madyson V. Weippert, Marie-Ève Labonté, Anthea K. Christoforou, Alena (Praneet) Ng, Mary R. L’Abbe

**Affiliations:** 1 Department of Nutritional Sciences, Temerty Faculty of Medicine, University of Toronto, Toronto, ON M5S 1A8, Canada; nadia.flexner@mail.utoronto.ca (N.F.); jodi.bernstein@mail.utoronto.ca (J.T.B.); madyson.weippert@mail.utoronto.ca (M.V.W.); anthea.christoforou@mail.utoronto.ca (A.K.C.);; 2 Centre Nutrition, Santé et Société (NUTRISS), Institute of Nutrition and Functional Foods (INAF), Laval University, Québec City, QC G1V 0A6, Canada; marie-eve.labonte@fsaa.ulaval.ca

**Keywords:** free sugars intake, food reformulation, modeling, diet-related NCD, macrosimulation model, food policy

## Abstract

Free sugars are a major source of calories in diets and contribute to the burden of many non-communicable diseases (NCDs). The World Health Organization (WHO) recommends reducing free sugars intake to less than 10% of total energy. This study aimed to estimate the number of diet-related NCD deaths which could be averted or delayed if Canadian adults were to reduce their calorie intake due to a systematic 20% reduction in the free sugars content in foods and beverages in Canada. We used the Preventable Risk Integrated ModEl (PRIME) to estimate the potential health impact. An estimated 6770 (95% UI 6184–7333) deaths due to diet-related NCDs could be averted or delayed, mostly from cardiovascular diseases (66.3%). This estimation would represent 7.5% of diet-related NCD deaths observed in 2019 in Canada. A 20% reduction in the free sugars content in foods and beverages would lead to a 3.2% reduction in calorie intake, yet an important number of diet-related NCD deaths could be averted or delayed through this strategy. Our findings can inform future policy decisions to support Canadians’ free sugars intake reduction, such as proposing target levels for the free sugars content in key food categories.

## 1. Introduction

Similarly to global trends, 88% of all deaths in Canada are due to non-communicable diseases (NCDs), with cardiovascular diseases (CVDs), cancer, and diabetes responsible for 59% of all deaths [1]. The prevalence of overweight and obesity has steadily increased in Canada in recent decades [2,3]. In 2018, 63.1% of adults were affected by either overweight (36.3%) or obesity (26.8%) [4]. Furthermore, Canadians who had obesity, as compared to those of normal weight, had a higher prevalence of type 2 diabetes (13.4% vs. 2.9%), high blood pressure (29.5% vs. 9.5%), and heart disease (6.0% vs. 2.7%) [4].

An unhealthy diet is one of the major preventable risk factors for a range of NCDs [5]. A healthy food environment can improve diets and, in turn, decrease the global burden of obesity and diet-related NCDs [6]. Food reformulation has been identified as one of the most cost-effective interventions for addressing the current burden of obesity and NCDs [7]. However, Canadian initiatives aiming to reduce the presence of nutrients of public health concern (e.g., saturated fat, sodium, and sugars) from the food supply have historically focused on reducing sodium and trans-fats, and have not specifically targeted sugars [8,9,10]. 

Free or added sugars are a major source of energy in diets—particularly from sugar-sweetened beverages (SSBs)—and excess consumption has been associated with an increased risk of obesity [11,12,13,14], type 2 diabetes [13,15,16], CVDs [13,17,18], and dental caries [13,19,20]. In 2015, the WHO published guidelines for sugars intake for adults and children, which strongly recommended reducing the intake of free sugars to under 10% of total energy (%TE) intake, with a conditional recommendation to further reduce intakes to under 5% in order to reduce the risk of associated adverse health outcomes [13]. According to the WHO, free sugars consist of “*monosaccharides and disaccharides added to foods and beverages by the manufacturer, cook or consumer, and sugars naturally present in honey, syrups, fruit juices and fruit juice concentrates*” [13]. 

In Canada, the government finalized sugars-related amendments to their Nutrition Facts table in 2016, including the grouping of sugar-based ingredients in the ingredient list and the declaration of a percent Daily Value (DV) for total sugars to be implemented by the end of 2022 [21,22]. However, the amendments did not include a declaration of free sugars content on nutrition labels, which makes efforts to monitor free sugars intake and changes in the amounts of this nutrient in the food supply challenging. Although free sugars reformulation target levels have not been proposed yet in Canada, implementing the recently published front-of-pack labeling (FOPL) regulations [23] may indirectly motivate the food industry to reformulate products, including the levels of free sugars. 

Public Health England introduced an initiative to reduce the added sugars content in key food categories by at least 20% by 2020, focusing on the top nine food categories that contribute the most to children’s sugars consumption [24]. A progress report on the strategy showed a 2.9% overall reduction in total sugars per 100 g of food between 2015 and 2018. Meanwhile, the average sugars content in sugary drinks that are subject to the UK Soft Drinks Industry Levy decreased by 28.8% in the same years [25]. Likewise, in the United States, the *National Salt and Sugar Reduction Initiative (NSSRI)* released a set of target levels to reduce the content of added sugars in 15 food and beverage categories by 10% by 2023, and by 20% (food) and 40% (beverages) by 2026 [26]. These food and beverage categories account for 70% of US child and youth sugars intakes, and it has been estimated that meeting 2023 and 2026 sugar target levels would reduce sugars intakes in this population by 7 and 21%, respectively [27]. Additionally, a microsimulation study predicted that achieving the NSSRI’s sugar reformulation targets could result in significant health gains (preventing 2.48 million CVD events, 0.49 million CVD deaths, and 0.75 million cases of diabetes), equity gains, and health care and societal cost savings (up to USD 160.88 billion) [28]. 

A recent study conducted by our group, using data from the Canadian Community Health Survey (CCHS) Nutrition 2015 [29], estimated that Canadians’ overall mean free sugars intake was 12.1%TE (56.2 g/day) [30], which greatly exceeds WHO recommended levels (i.e., less than 10%TE) [13]. This result is consistent with other analyses in the Canadian population [31,32,33]. The study also found that reducing free sugars intake to at least the upper recommended level (10%), through a systematic 20% reduction of free sugars content in foods and beverages, would lead overall to a 3.2% reduction of daily calorie intake (−60 calories) [30]. Considering that reducing the free sugars content in food and beverages may not necessarily result in an equivalent reduction in calories [34,35], results from this study suggest how calorie intakes from reducing the free sugars content in food and beverages in the Canadian food supply would change [30]. However, the potential health impact from these changes in calorie consumption derived from reducing free sugars intake remains unknown in the Canadian context and less studied globally.

To support the decision-making process, policymakers rely more than ever on health and economic analysis, using simulation modeling methods as a tool for policy support and prioritization [36,37]. Moreover, given the evident challenges of performing traditional epidemiological research methods (such as cohort studies, randomized controlled trials, or natural experiments) to measure the health impact of public health policies, simulation modeling methods are a suitable and strategic tool with which to estimate the impact of a policy before actual policy adoption and implementation [38,39,40]. For the current study we used the Preventable Risk Integrated ModEl (PRIME), a comparative risk assessment model [40], to estimate potential health gains. 

To the best of our knowledge, no study has estimated the potential health benefits from changes in calorie intake as a result of food product reformulation in Canada. Building on our previous work, the objective of the present study was to estimate the number of diet-related NCD deaths which could be averted or delayed if Canadian adults were to reduce their calorie intake as a result of a systematic 20% reduction in the free sugars content in foods and beverages in Canada.

## 2. Materials and Methods

The current study employed PRIME, a cross-sectional NCD scenario model, to estimate the number of diet-related NCD deaths in Canada that could be averted or delayed due to reducing consumption of calories derived from reducing free sugars intake. The data requirements of PRIME include the following age- and sex-specific sets of data from the population under study: (1) estimates of the number of individuals in the population; (2) estimates of the annual number of deaths from each NCD of interest; (3) population distribution of behavioral risk factors (baseline scenario) and the counterfactual distribution of behavioral risk factors being studied (counterfactual scenario). Additionally, the age- and sex-specific mean height and body mass index (BMI) of Canadians (≥19 y) were estimated and included in the model. Details of the policy scenario modeled and sources of data used in this study are described below.

### 2.1. Health Impact Modeling

PRIME is a macrosimulation NCD scenario model that has been applied to study different scenarios of public health strategies, and it has been used in several country contexts [40], including in the Canadian population [41,42,43,44]. PRIME estimates the impact of changes in the distribution of behavioral risk factors at the population level on NCD mortality [40]. It is an open-access and user-friendly NCD scenario modeling tool that is available from its authors at the University of Oxford upon request and was recently made accessible through the WHO Regional Office for Europe web portal [45]. PRIME studies twelve behavioral risk factors, including alcohol consumption, smoking, physical activity, and diet (energy, fruits and vegetables, fiber, salt, total fat, saturated fat, unsaturated fat, and cholesterol), as well as twenty-four health outcomes, including CVDs, cancers, diabetes, kidney disease, liver disease, and chronic obstructive pulmonary disease. Twenty-three of these outcomes are diet-related NCDs [40]. The impact on the health outcomes due to a change in one or more behavioral risk factors is modelled directly or through one of three intermediate risk factors: blood pressure, serum blood cholesterol, or BMI [40]. The model parameterizes the links between the behavioral risk factors and NCD mortality using meta-analyses taken from epidemiological studies [40]. 

PRIME does not include the consumption of free sugars as a behavioral risk factor, but the effects can be mediated through changes in calories as a consequence of modifying free sugars consumption. As calories are known to moderate many of the effects of sugars on chronic disease outcomes [11,12,13,15,16,17,18], the current study aimed to estimate deaths that would be prevented or delayed due to changes in BMI (intermediate risk factor) as a result of changes in population calorie intakes. The following section details how the baseline and counterfactual scenarios included in the model were established.

### 2.2. Dietary Data Collection 

For the baseline scenario, we considered Canadians’ actual mean calorie intakes stratified by Dietary Reference Intakes (DRI) age–sex groups. For the counterfactual scenario, we included Canadians’ estimated mean calorie intakes after modeling a systematic 20% reduction in the free sugars content in foods and beverages consumed by Canadians, which have already been published by our research group [30,35]. Briefly, data from CCHS Nutrition 2015 [29] were used to estimate Canadians’ calorie intakes. CCHS Nutrition 2015 is a nationally representative, cross-sectional food and nutrition survey that uses a 24 h dietary recall conducted via computer-assisted in-person interviews by trained personnel. It collected data on 20,487 individuals, with an additional recall conducted via telephone on 35% of the sample. This analysis included the entire CCHS Nutrition 2015 sample, excluding pregnant and breastfeeding women and respondents with null intakes, which left a total of 20,176 participants included in the study. 

CCHS Nutrition 2015 does not include data on free sugars intake levels; thus, free sugars and the corresponding calories of foods and beverages were estimated using the Food Label Information Program (FLIP) 2017 database [8]. FLIP 2017, developed by the University of Toronto, contains Canadian food package label information by brand name for the main food and beverages sold in Canada [8]. FLIP 2017 contains nutritional information for nearly 17,700 unique food and beverage products [8]. The information collected in FLIP includes the Nutrition Facts Table (NFt), an ingredients list, the product price, and photos of all sides of the product packaging [8], among other information. FLIP 2017 also contains the free sugars content of products, calculated using a modified version of the University of Toronto’s Free Sugar Algorithm—a step-by-step methodology for estimating free sugars using the information available on food packaging [35,46]. 

#### 2.2.1. Baseline Scenario: Canadians’ Actual Calorie Intake

For the baseline scenario, we used Canadian age- and sex-specific calorie intakes (Table 1). Canadian free sugars and calorie intakes (mean, SE) were calculated by merging two sets of databases, the free sugars and calorie content of foods and beverages estimated in FLIP, and the Food and Ingredient Details (FID) file utilized in CCHS Nutrition 2015 (24 h recall). This procedure was conducted by matching food products from FLIP 2017 with similar CCHS Nutrition 2015 food profiles; these methods have been previously published [30,47].

#### 2.2.2. Counterfactual Scenario: Canadians’ Calorie Intake after Reformulation of the Food Supply to Contain 20% Less Free Sugars

For the counterfactual scenario, we included the resultant Canadian age- and sex-specific calorie intake after modeling a systematic 20% reduction in the free sugars levels of food and beverages and the consequent changes in calorie consumption (Table 1) [30,35]. The rationale behind the proposed average reduction of 20% lies, in part, on an average (range 15–25%) of actual food reformulation (i.e., lower in sugars) observed in some food categories between 2013 and 2017 [34], the Public Health England sugar reduction target [24], and the percentage reduction needed to lower intakes from 12.1% to 10.0%TE (approximately a 20% reduction). Previous research from our group reported that most products reformulated to be lower in sugars are not necessarily lower in calories (e.g., due to sugars being replaced by other components such as fat or starch). On average, a 19% reduction in sugars was observed among the products that were reformulated to be lower in sugars—mainly beverages and desserts and sweets [34]. Thus, the free sugars levels for all relevant food categories (bakery; beverages; cereals and grains; dairy and alternatives; desserts; fats and vinegars; fish and seafood; fruits; mixed dishes; sauces and dips; snacks; soups; sugars and sweets; and vegetables) were systematically reduced by 20%, and calorie and other nutrient levels were adjusted to mirror actual changes in calories and nutrients observed in similar products reformulated to be lower in sugars between 2013 and 2017 [35]. The nutrient composition of reformulated products (free sugars and calorie levels) was then linked with CCHS Nutrition 2015 24 h recall data to estimate calorie intakes under the reformulation scenario [30]. 

Canadian free sugars and calorie intakes, stratified by DRI age–sex groups, under the actual (baseline) and counterfactual (reformulation) scenario were estimated using both available days of 24 h recalls from CCHS Nutrition 2015. Usual nutrient intake distributions were determined using the National Cancer Institute (NCI) method [48]. Balanced repeated replication was used to estimate all standard errors using bootstrap weights provided by Statistics Canada. To allow for representative estimates, all analyses were weighted using sampling weights provided by Statistics Canada. Analyses for adults (≥19 y) were adjusted for age and dietary misreporting status [30].

### 2.3. Population Demographics

In the present study, population demographics and the most recent available data (2019) on mortality associated with CVDs (ischemic heart diseases, cerebrovascular disease, heart failure, aortic aneurysm, pulmonary embolism, rheumatic heart disease, and hypertensive disease), cancers (lip, oral cavity and pharynx, oesophagus, stomach, bronchus and lung, pancreas, colorectum, breast, endometrium, gallbladder, kidney, bladder, liver, and cervix), diabetes, chronic renal failure, and liver disease—stratified by sex and five-year age bands—were obtained from the publicly available Statistics Canada CANSIM tables [49,50,51,52,53,54]. It was assumed that there were no major changes in Canadian calorie intakes between 2015 and 2019, considering previous estimations [55]. Mortality data were based on the WHO International Classification of Diseases 10 (ICD 10) [56]. Detailed information and data sources are available in the Supplemental Materials.

### 2.4. Statistical Analyses

For this study, mean height and BMI were calculated for the same sample included in both the baseline and counterfactual scenario (CCHS Nutrition 2015). Previous research on both the 2004 and the 2015 cycles of CCHS Nutrition has shown that adult women tend to underreport their weight, and adult men tend to overreport their height. To mitigate the introduction of these systematic biases into the analyses, BMI correction factors provided by Statistics Canada were used in this manuscript to generate respondents’ BMI and height from self-reported values, if measured values were not available [57]. The final mean BMI and mean heights for the sample were generated from both these corrected and measured values (Table 1). All data analyses were conducted using SAS 9.4, and a *p*-value < 0.05 was considered statistically significant. 

All required data were inputted into PRIME. The model estimated changes in the annual number of deaths attributable to the diet-related NCDs under study between the baseline and the counterfactual scenario. Monte Carlo analysis, built into PRIME, was performed at 10,000 iterations to estimate 95% uncertainty intervals (UIs) around the results (based on the 2.5th and 97.5th percentiles). This allowed the epidemiological parameters to vary randomly according to the distributions reported in the literature [40]. 

## 3. Results

The PRIME estimated that overall, 6770 (95% UI 6184–7333) diet-related NCD deaths could be averted or delayed by reducing Canadian adults’ calorie intake as a result of a systematic 20% reduction in the free sugars content in packaged foods and beverages (men 3704 [95% UI 3394–3988]; women 3082 [95% UI 2692–3429]). From total deaths that could be prevented or delayed, 66.3% (4491 [95% UI 3999–4953]) are related to CVDs, followed by diabetes 14.1% (954 [95% UI 734–1083]), cancers 11.5% (781 [95% UI 609–958]), liver disease 5.2% (351 [95% UI 188–471]), and chronic renal failure 3.2% (218 [95% UI 85–307]) (Table 2).

When looking individually at each disease, we observed the greatest sex differences for ischemic heart disease, where the difference between men (1593 [95% UI 1401–1774]) and women (806 [95% UI 534–1047]) almost doubles. Furthermore, 33.9% of potential deaths averted or delayed would be in people under 75 years old, affecting significantly more men (1545 [95% UI 1415–1661]) than women (752 [95% UI 659–834]) (Table 2).

The model estimated that the changes in calorie intakes between the baseline and counterfactual scenario would impact 13 of the 23 diet-related NCDs included in this analysis. To put our results in context, in 2019, there were a total of 90,035 deaths due to these 13 diet-related NCDs in Canada (CVDs: ischemic heart disease, cerebrovascular disease, heart failure, and hypertensive disease; diabetes; cancers: pancreas, colorectum, breast, endometrium, gallbladder, and kidney; chronic renal failure; and liver disease) [50,51,52,53,54]; our results would represent 7.5% (8.2% in men, 6.9% in women) fewer NCD deaths, which could have been averted or delayed in Canada if changes in calorie intakes deriving from free sugars had occurred (reformulation scenario)—an estimated 14.6% from diabetes, 10.3% from chronic renal failure, 9.6% from liver disease, 8.1% from CVDs, and 3.5% from cancers (Table 2).

## 4. Discussion

The current study, to the best of our knowledge, is the first modeling study to estimate the potential health impact of reducing calorie intakes through a systematic 20% reduction in the free sugars content in food and beverages in the Canadian food supply. As a result of this simulated food reformulation scenario, Canadians’ overall mean intake of free sugars would fall from 56.2 g/day (12.1%TE) to 44.9 g/day (10.0%TE), resulting in a reduction in overall calorie intake of 60 calories (−3.2%) [30]. If such a level of food reformulation occurred—and assuming food choices would not vary regardless of changes in the free sugars content in food and beverages—6770 (95% UI 6184–7333) deaths due to diet-related NCDs could be averted or delayed in Canada. To put this in context, these estimates would represent 7.5% of diet-related NCD deaths observed in 2019 in Canada. Most of the deaths averted or delayed would have occurred in people older than 75 years of age. 

The current literature demonstrates that most of the adverse health effects associated with an excess intake of free sugars are mediated to some extent by their energy contribution [12,16,17,58]. Therefore, reformulating food products to contain less free sugars should also aim to reduce energy content as much as possible [24,34]. Evidence from other countries shows that the implementation of a mix of population-wide interventions can improve diets. Specifically, government-led initiatives such as implementing food reformulation targets, FOPL, and SSB taxation could lead to reformulated foods lower in free sugars, changes in consumers’ purchasing behavior, health gains, and healthcare cost savings [59,60], as results from the present study also suggest. 

In Canada, a previous modeling study estimated that, over 25 years, 7874 (95% UI 6630, 9118) deaths could be postponed by implementing a 20% SSB tax [61]. The simulated SSB tax was estimated to reduce Canadian adults’ per capita daily energy intake by 21 kcal/day for men and 13 kcal/day for women [61]. Furthermore, our research group previously estimated that by replacing, whenever possible, foods that would carry a red traffic light label (TLL) with foods that would not carry a red TLL (following thresholds applied in the UK system) 11,715 deaths from diet-related NCDs could have been prevented or postponed in Canada, of which 90% (n = 10,490) of the estimated lives saved were attributable to reductions in calorie intake, compared to 6% from a reduction in sodium intake and 6% from a reduction in fat intake (total and saturated fatty acids) [42]. Results from this earlier study therefore suggest that most of the health gains would come from reductions in mean energy intakes, which would be reduced on average by 122 kcal/day and 90 kcal/day for men and women, respectively. Although avoiding foods with a red TLL altogether would be an idealistic scenario that would require changes in consumer behaviour, there is evidence of promising changes in household beverage purchases as a result of implementing a mandatory FOPL [59]. For instance, in Chile, an evaluation of the comprehensive Chilean FOPL law, in an 18-month window before and after implementation, found a reduction of nearly 24% in purchases of beverages carrying a ‘high in’ FOPL [59]. Moreover, a year after the implementation of the Chilean FOPL law, there was a significant decrease in the proportion of food products displaying any ‘high in’ warning label (from 51% to 44%), with the most frequent reductions observed in the proportion of food products carrying a sugar or sodium ‘high in’ warning label [62]. Specifically, the proportion of products ‘high in’ sugars went from 80% to 60% for food categories such as beverages, milk and milk-based drinks, breakfast cereals, sweet baked products, and sweet and savory spreads [62]. Additionally, a recent economic evaluation suggested that FOPL reduces sugars consumption and, consequently, can significantly reduce dental caries and the caries-related economic burden in the German population, preventing nearly 2.4 million caries lesions and reducing dental treatment costs by up to 3.2% over a 10-year period [63]. All in all, the evidence suggests promising health gains, changes in consumers’ behavior, and food product reformulation towards reducing sugars content as a result of implementing a mandatory FOPL. Future evaluations of the recently approved FOPL regulations in Canada [23] will provide evidence on the impact of this policy on consumers’ food purchasing behavior and industry-driven food product reformulation, including sugars. 

In Canada, evidence demonstrates that, although there has been little effort to reduce overall sugars content through food reformulation between 2013 and 2017, some foods still presented significant reformulation of their free sugars content (with a median decrease of 19%) [34]. Beverages and desserts had the highest proportion of products showing a reduction in their sugars levels, corresponding to −22.2% and −20.3%, respectively [34]. However, for some products, the reduction in sugars levels does not necessarily translate into lower energy content, due to increased starches [34]. Given the different functions sugar serves in foods, other than influencing taste—such as its contribution to smell, texture, colour, bulk, viscosity, crystallization, stabilization, other chemical functions, and preservation [64,65,66,67]—food product reformulation to contain less free sugars might not be as straightforward as decreasing or removing the nutrient from foods, as is the case with sodium. Instead, other ingredients might need to be added to foods to compensate for the functional components of free sugars (e.g., fats and starches), as evidence suggests [30,34,35]. This could have unintended consequences, such as a reduction in sugar not leading to an equivalent reduction in food caloric content, or even leading to an increased caloric content. In the Canadian food supply, food categories with high added and free sugars content include desserts and sweets, breakfast cereals, baked products, beverages, and snacks [31]. The two main food groups that contribute the most to the added, free, and total sugars intake in the diet of Canadians are desserts and sweets (67.3%, 57.5%, and 41.1%, respectively) and beverages (17.4%, 17.5%, and 12.6%, respectively) [31]. This could suggest that most of the health gains presented in this study could come from reductions in the free sugars content in these two food categories. 

The present study has strengths and limitations. Estimated intakes in the counterfactual scenario were calculated using nutrient data derived from a reformulation scenario [35] that was based on the current Public Health England free sugars reduction target of 20% [24], but, more importantly, the counterfactual scenario was based on actual observed changes in nutrient levels and calories from foods in the Canadian food supply that were reformulated to be lower in sugars between 2013 and 2017 [30,34,35].

The baseline and counterfactual intake scenarios were estimated from a nationally representative sample of the Canadian population (CCHS Nutrition 2015) [30]. Given the nature of the survey, there could be issues with misreporting; however, CCHS Nutrition 2015 used the Automated Multiple Pass Method to minimize misreporting bias [29]. Moreover, the NCI method was used to estimate usual intakes, which is particularly useful to adjust for within and between-person variations [48]. To calculate the free sugars content in foods and beverages and free sugars intake, a representative database of foods and beverages sold in Canada was used (FLIP 2017) [8].

Furthermore, our counterfactual scenario takes into account substitution effects at the food composition level (i.e., changes in calorie and nutrient levels from reformulated products between 2013 and 2017) [34]. However, we did not consider any substitution effect or caloric compensation at the consumer level and assumed that consumers’ food choices would remain the same. Additionally, we modeled the effects on diet-related NCD deaths specifically from reducing calorie intakes as a result of a modeled 20% reduction in the free sugars content in food and beverages in the Canadian food supply; however, estimated health effects could have also been observed through calorie reductions resulting from changes in other macronutrients. Nevertheless, in our counterfactual scenario, the reductions in calories through dietary reductions in free sugars is a function of the nature of the food (i.e., foods high in free sugars) and the extent of free sugars present in those foods, as well as the dietary habits and patterns of the Canadian population (i.e., the amount of these foods the population is eating). Given the nature of this study, the data do not allow for the investigation of causal relationships.

To estimate the number of diet-related NCD deaths that could be averted or delayed in Canada, we used PRIME, which uses relative risks from robust meta-analyses, and which has been widely used in different countries [40], including Canada [41,42,43,44]. However, PRIME is a cross-sectional NCD scenario model, which means that the model does not incorporate the effect of time lag between the disease outcome and the exposure [40]. Therefore, it is not clear when the estimates predicted by the model would be expected to be achieved. Additionally, PRIME does not investigate morbidity that could be prevented from changes in NCD behavioral risk factors; thus, this aspect was not captured in the present study. The strengths and limitations of PRIME have been previously documented [40]. 

Finally, implementing target levels for the free sugars content in key food categories in addition to other proposed initiatives in Canada that aim to improve the food environment, such as FOPL and restrictions on food marketing to children [23,68], could potentially improve the diet of Canadians and prevent or delay an important number of diet-related NCDs. This is particularly important to protect vulnerable populations such as children and adolescents, whose free sugars intake seems to be higher than in other age groups [30,61,69]. If strategies to reduce the free sugars content in packaged foods are implemented in Canada, robust and independent monitoring and evaluations will be required to understand changes in consumers’ food purchasing behavior and intakes, as well as changes in the food supply (e.g., the introduction of new products; an increased use of non-caloric sweeteners; and the potential replacements for free sugars, such as fats or starches).

## 5. Conclusions

This study provides evidence of the potential health gains from reducing intakes of calories from free sugars as a result of reducing the free sugars content in foods and beverages in Canada. Our results show that a 20% reduction in the free sugars content in foods and beverages could lead to a 3.2% reduction in calorie intake, and 7.5% of diet-related NCD deaths could be averted or delayed in Canada. Our findings can inform future policy decisions to support Canadians’ free sugars intake reduction, such as proposing target levels for the free sugars content in key food categories. 

## Figures and Tables

**Table 1:** Mean calorie intake (kcal/day) for baseline and reformulation scenario (a systematic reduction in free sugars content in packaged foods and beverages by 20%).

	Age	*n*	Mean Kcal (SE) “Baseline” ^1^	Reformulation Scenario Mean Kcal (SE) “Counterfactual” ^2^	Difference (Kcal and %)	Mean Height (m) ^3^	Mean BMI (SE) ^3^
Total	1+	20,176	1858 (12)	1798 (11)	60 (3.2%)	–	–
Male	19–30	882	2023 (44)	1956 (43)	67 (3.3%)	1.76	25.69 (0.36)
	31–50	2077	2037 (26)	1972 (25)	65 (3.2%)	1.76	28.41 (0.23)
	51–70	2246	2054 (29)	1991 (29)	63 (3.1%)	1.74	28.98 (0.21)
	71+	1246	2091 (26)	2030 (26)	61 (2.9%)	1.72	27.96 (0.24)
Female	19–30	897	1515 (35)	1462 (34)	53 (3.5%)	1.64	25.02 (0.50)
	31–50	2288	1567 (22)	1515 (21)	52 (3.3%)	1.63	26.72 (0.25)
	51–70	2420	1642 (19)	1590 (19)	52 (3.2%)	1.61	27.74 (0.21)
	71+	1556	1658 (24)	1608 (24)	50 (3.0%)	1.58	27.35 (0.24)

Data sources. ^1^. Baseline mean calorie intake: CCHS Nutrition 2015 [29]. ^2^. Counterfactual scenario mean calorie intake after systematically reducing free sugars content in foods and beverages by 20%: FLIP 2017 [8] and CCHS Nutrition 2015 [29]. ^3^. BMI and height estimates: CCHS Nutrition 2015 [29].

**Table 2:** Estimated number of deaths that could be averted or delayed if Canadians reduced their consumption of calories derived from free sugars intake as a result of reducing free sugars content in packaged food products by 20%—by cause of death (95% UI).

*Cause of Death (ICD-10 Code)* ^1^	*Total Mean (95% UI)* ^2^	*%*	*Men Mean (95% UI)* ^2^	*%*	*Women Mean (95% UI)* ^2^	*%*
**Cardiovascular diseases**	**4491 (3999, 4953)**	**66.3**	**2500 (2250, 2742)**	**67.5**	**2000 (1648, 2313)**	**64.9**
Ischemic heart disease (I20–25)	2392 (2055, 2695)	35.3	1593 (1401, 1774)	43.0	806 (534, 1047)	26.2
Cerebrovascular disease (I60–69)	791 (555, 1021)	11.7	341 (241, 438)	9.2	452 (308, 585)	14.7
Heart failure (I50)	766 (480, 978)	11.3	338 (209, 435)	9.1	426 (258, 539)	13.8
Hypertensive disease (I10–15)	559 (432, 636)	8.3	234 (184, 267)	6.3	327 (253, 371)	10.6
*Actual deaths in 2019*	*55,662*		*29,219*		*26,443*	
*% deaths averted or delayed* ^3^	*8.1*		*8.6*		*7.6*	
**Diabetes (E11, E14)**	**954 (734, 1083)**	**14.1**	**528 (414, 598)**	**14.3**	**429 (329, 488)**	**13.9**
*Actual deaths in 2019*	*6536*		*3664*		*2872*	
*% deaths averted or delayed* ^3^	*14.6*		*14.4*		*14.9*	
**Cancer**	**781 (609, 958)**	**11.5**	**351 (265, 435)**	**9.5**	**431 (339, 520)**	**14.0**
Pancreas (C25)	140 (28, 256)	2.1	71 (13, 126)	1.9	69 (13, 124)	2.2
Colorectum (C18–C20)	357 (236, 470)	5.3	191 (130, 251)	5.2	165 (111, 218)	5.4
Breast (C50)	37 (−9, 82)	0.5	0	0	38 (−6, 83)	1.2
Endometrium (C54.1)	103 (76, 124)	1.5	0	0	103 (76, 124)	3.3
Gallbladder (C23)	14 (10, 19)	0.2	5 (4, 7)	0.1	9 (6, 12)	0.3
Kidney (C64)	131 (105, 156)	1.9	84 (67, 100)	2.3	47 (38, 56)	1.5
*Actual deaths in 2019*	*22,060*		*8777*		*13,283*	
*% deaths averted or delayed* ^3^	*3.5*		*4.0*		*3.2*	
**Chronic renal failure (N18)**	**218 (85, 307)**	**3.2**	**109 (48, 156)**	**2.9**	**107 (45, 151)**	**3.5**
*Actual deaths in 2019*	*2118*		*1102*		*1016*	
*% deaths averted or delayed ^3^*	*10.3*		*9.9*		*10.5*	
**Liver disease (K70, K74)**	**351 (188, 471)**	**5.2**	**227 (127, 303)**	**6.1**	**124 (59, 171)**	**4.0**
*Actual deaths in 2019*	*3659*		*2364*	*9.6*	*1295*	
*% deaths averted or delayed* ^3^	*9.6*		*9.6*		*9.6*	
***Total deaths prevented under 75***	***2293 (2092, 2483)***	***33.9***	***1545 (1415, 1661)***	***41.7***	***752 (659, 834)***	***24.4***
**Total deaths averted or delayed**	**6770 (6184, 7333)**	**100**	**3704 (3394, 3988)**	**100**	**3082 (2692, 3429)**	**100**
*Total actual deaths from diseases under study*	*90,035*		*45,126*		*44,909*	
*% deaths averted or delayed* ^3^	*7.5*		*8.2*		*6.9*	

^1^. WHO International Statistical Classification of Diseases and Related Health Problems, Tenth Revision [56]. ^2^. 95% UI are based on 10,000 iterations of Monte Carlo analysis built into PRIME [40]. ^3^. Percentage of actual deaths in Canada (2019) attributable to the diet-related NCDs under study. Note: total deaths averted or delayed represent less than the sum of the individual diet-related NCD mortality causes given that double counting has been accounted for in PRIME during the modeling process. The same applies to the sum of CVDs and cancers.

## Data Availability

Canadian population demographics and data on mortality associated with diet-related NCDs (CVDs, diabetes, cancer, chronic renal failure, and liver disease)—stratified by sex and five-year age band—were obtained from the publicly available Statistics Canada CANSIM tables (2019). https://www150.statcan.gc.ca/t1/tbl1/en/tv.action?pid=1710000501 (accessed on 4 January 2021), https://www150.statcan.gc.ca/t1/tbl1/en/tv.action?pid=1310014201 (accessed on 4 January 2021), https://www150.statcan.gc.ca/t1/tbl1/en/tv.action?pid=1310014401 (accessed on 4 January 2021), https://www150.statcan.gc.ca/t1/tbl1/en/tv.action?pid=1310014701 (accessed on 4 January 2021), https://www150.statcan.gc.ca/t1/tbl1/en/tv.action?pid=1310015101 (accessed on 4 January 2021), https://www150.statcan.gc.ca/t1/tbl1/en/tv.action?pid=1310014801 (accessed on 4 January 2021).

## Supplementary Materials

The following supporting information can be downloaded at: https://www.mdpi.com/article/10.3390/nu15081835/s1, Table S1. Age- and sex-specific estimates of the Canadian population, 2019; Table S2. Age- and sex-specific estimates of the annual number of diet related NCD deaths in Canada, 2019.
