# Supplementary material for: How Many Diet-Related Non-Communicable Disease Deaths Could Be Averted or Delayed If Canadians Reduced Their Consumption of Calories Derived from Free Sugars Intake? A Macrosimulation Modeling Study
Source: Nutrients. 2023 Apr 11;15(8):1835. doi: 10.3390/nu15081835 (PMC10140857; doi:10.3390/nu15081835)
Supplement: Supplementary file 1 [file nutrients-15-01835-s001.zip › nutrients-2308811-supplementary.pdf]

Supplemental Materials

How Many Diet-Related Non-Communicable Disease Deaths Could Be Averted or Delayed If Canadians Reduced Their Consumption of Calories Derived from Free Sugars Intake? A Macrosimulation Modeling Study

Nadia Flexner [1], Jodi T. Bernstein [1], Madyson V. Weippert [1], Marie-Ève Labonté [2], Anthea K. Christoforou [1], Alena (Praneet) Ng [1] and Mary R. L'Abbe [1,*]

[1] Department of Nutritional Sciences, Temerty Faculty of Medicine, University of Toronto, Toronto, ON M5S 1A8, Canada
[2] Centre Nutrition, Santé et Société (NUTRISS), Institute of Nutrition and Functional Foods (INAF), Laval University, Québec City, QC G1V 0A6, Canada
* Correspondence: mary.labbe@utoronto.ca

Contents

Table S1. Age- and sex-specific estimates of the Canadian population, 2019 2

Table S2. Age- and sex-specific estimates of the annual number of diet related NCD deaths in Canada, 2019 3

Table S1. Age- and sex-specific estimates of the Canadian population, 2019 [1].

Age	Male	Female
**20-24**	1,292,739	1,182,807
**25-29**	1,353,893	1,272,165
**30-34**	1,319,176	1,285,925
**35-39**	1,288,783	1,292,390
**40-44**	1,198,765	1,223,278
**45-49**	1,190,901	1,206,950
**50-54**	1,246,625	1,257,812
**55-59**	1,368,185	1,383,192
**60-64**	1,236,748	1,276,958
**65-69**	1,017,348	1,080,436
**70-74**	817,561	890,019
**75-79**	543,317	621,481
**80-84**	348,047	440,404
**85+**	304,616	531,841

Table S2. Age- and sex-specific estimates of the annual number of diet related NCD deaths in Canada, 2019 [2–6].

Males	20-24	25-29	30-34	35-39	40-44	45-49	50-54	55-59	60-64	65-69	70-74	75-79	80-84	85+
I60-I69: Cerebrovascular diseases	3	7	14	22	30	72	101	190	280	452	641	829	1,018	2,288
I20-I25: Ischaemic heart diseases	2	8	17	65	122	269	573	1,049	1,631	1,999	2,324	2,394	2,691	5,626
C00-C14: Lip, oral cavity and pharynx	2	2	3	4	11	28	53	110	158	158	185	131	86	126
C15: Oesophagus	0	0	3	9	17	33	76	158	238	281	273	232	203	198
C16: Stomach	0	4	5	7	12	22	48	91	130	162	175	175	172	241
C34: Bronchus and lung	2	3	4	12	26	57	211	589	1,133	1,590	1,946	1,815	1,440	1,551
C25: Pancreas	0	3	4	3	13	39	92	200	334	374	475	429	321	431
C18-20: Colorectum	3	5	22	34	61	104	172	318	440	564	680	613	703	1,031
C50: Breast	-	-	-	-	-	-	-	-	-	-	-	-	-	-
C54.1: Endometrium	-	-	-	-	-	-	-	-	-	-	-	-	-	-
C23: Gallbladder	0	0	0	0	1	3	4	6	5	18	15	14	9	19
C64: Kidney	0	0	1	6	8	14	49	89	129	163	191	151	146	225
I10-I15: Hypertensive disease	0	0	5	6	14	27	58	97	117	136	156	167	244	620
E11, E14: Diabetes	5	4	13	16	42	64	100	225	294	390	538	526	522	923
C67: Bladder cancer	0	0	1	2	2	11	16	39	90	136	197	215	289	545
C22: Liver cancer	1	3	3	10	15	15	67	180	291	367	364	308	246	229
C53: Cervix cancer	-	-	-	-	-	-	-	-	-	-	-	-	-	-
K70, K74: Liver disease	0	13	17	35	62	115	188	329	363	417	307	229	172	117
I50: Heart failure	0	2	1	0	9	4	35	47	95	119	232	293	480	1,534
I71: Aortic aneurysm	1	1	6	11	12	22	32	41	79	90	141	104	144	260
I26: Pulmonary embolism	2	3	2	1	4	10	15	27	22	25	47	43	31	59
I05-09: Rheumatic heart disease	0	0	1	2	4	3	5	6	10	9	29	34	35	75
N18: Chronic renal failure	1	1	0	2	5	8	7	24	42	72	97	152	183	508

Females	20-24	25-29	30-34	35-39	40-44	45-49	50-54	55-59	60-64	65-69	70-74	75-79	80-84	85+
I60-I69: Cerebrovascular diseases	3	7	16	19	26	56	89	138	221	322	516	746	1,044	4,494
I20-I25: Ischaemic heart diseases	1	1	3	14	32	70	142	263	511	717	1,067	1,216	1,845	7,230
C00-C14: Lip, oral cavity, and pharynx	1	0	1	2	4	12	16	25	47	43	59	63	50	91
C15: Oesophagus	0	0	1	1	3	6	13	28	53	64	75	82	56	99
C16: Stomach	0	2	5	5	15	14	29	50	49	80	94	106	112	184
C34: Bronchus and lung	0	1	4	12	36	74	187	612	1,009	1,326	1,690	1,596	1,242	1,523
C25: Pancreas	0	1	4	5	12	32	68	154	221	298	374	376	385	565
C18-20: Colorectum	1	1	10	22	56	69	136	192	266	334	430	501	549	1,285
C50: Breast	1	2	42	62	119	193	350	454	508	570	624	585	579	1,179
C54.1: Endometrium	1	0	1	2	9	17	24	66	117	139	164	119	117	128
C23: Gallbladder	0	0	0	0	1	0	7	5	10	17	24	27	24	38
C64: Kidney	0	0	1	1	3	8	16	42	41	63	75	79	91	189
I10-I15: Hypertensive disease	0	0	0	2	7	14	15	27	61	92	124	166	259	1,418
E11, E14: Diabetes	2	3	7	14	14	25	56	112	155	249	308	342	428	1,157
C67: Bladder cancer	0	0	0	1	5	7	11	33	21	52	67	95	99	281
C22: Liver cancer	1	1	0	5	13	15	35	75	117	142	160	152	180	237
C53: Cervix cancer	2	3	12	25	37	36	42	60	46	44	38	29	20	39
K70, K74: Liver disease	2	6	17	28	32	65	104	157	172	203	156	141	95	117
I50: Heart failure	0	2	1	2	3	4	6	26	43	77	159	243	415	2,465
I71: Aortic aneurysm	0	0	1	1	2	3	7	7	18	38	60	84	109	284
I26: Pulmonary embolism	3	2	1	6	8	6	11	18	22	36	45	44	40	115
I05-09: Rheumatic heart disease	1	1	1	2	3	3	6	9	12	29	31	51	63	190
N18: Chronic renal failure	0	4	3	2	1	2	15	14	25	49	79	114	156	552

References

1. Statistics Canada. Table 17-10-0005-01 Population estimates on July 1st, by age and sex. Available at: https://www150.statcan.gc.ca/t1/tbl1/en/tv.action?pid=1710000501. Accessed January 4, 2021.
2. Statistics Canada. Table 13-10-0142-01 Deaths, by cause, Chapter II: Neoplasms (C00 to D48). Available at: https://www150.statcan.gc.ca/t1/tbl1/en/tv.action?pid=1310014201. Accessed January 4, 2021.
3. Statistics Canada. Table 13-10-0144-01 Deaths, by cause, Chapter IV: Endocrine, nutritional and metabolic diseases (E00 to E90). Available at: https://www150.statcan.gc.ca/t1/tbl1/en/tv.action?pid=1310014401. Accessed January 4, 2021.
4. Statistics Canada. Table 13-10-0147-01 Deaths, by cause, Chapter IX: Diseases of the circulatory system (I00 to I99). Available at: https://www150.statcan.gc.ca/t1/tbl1/en/tv.action?pid=1310014701. Accessed January 4, 2021.
5. Statistics Canada. Table 13-10-0151-01 Deaths, by cause, Chapter XIV: Diseases of the genitourinary system (N00 to N99). Available at: https://www150.statcan.gc.ca/t1/tbl1/en/tv.action?pid=1310015101. Accessed January 4, 2021.
6. Statistics Canada. Table 13-10-0148-01 Deaths, by cause, Chapter XI: Diseases of the digestive system (K00 to K93). Available at: https://www150.statcan.gc.ca/t1/tbl1/en/tv.action?pid=1310014801. Accessed January 4, 2021.
